# Ibuprofen versus mecillinam for uncomplicated cystitis - a randomized controlled trial study protocol

**DOI:** 10.1186/s12879-014-0693-y

**Published:** 2014-12-17

**Authors:** Ingvild Vik, Marianne Bollestad, Nils Grude, Anders Bærheim, Sigvard Mölstad, Lars Bjerrum, Morten Lindbæk

**Affiliations:** Antibiotic Centre of Primary Care, Department of General Practice, Institute of Health and Society, University of Oslo, Blindern, Oslo, 0318 Norway; Department of General Practice, Oslo Accident and Emergency Out Patient Clinic, Storgata 40, Oslo, 0182 Norway; Department of Medical Microbiology, Vestfold Hospital Trust, Tønsberg, 3103 Norway; Department of Global Public Health and Primary Care, University of Bergen, Bergen, 5018 Norway; Department of Clinical Sciences, Malmoe, General Practice, University of Lund, Lund, 221 00 Sweden; Department of Public Health, University of Copenhagen, Copenhagen, 1014 Denmark; Division of Medicine, Stavanger University Hospital, Stavanger, 4068 Norway

**Keywords:** Cystitis, NSAID, Antibiotics, Ibuprofen, Mecillinam, General practice

## Abstract

**Background:**

Although uncomplicated cystitis is often self-limiting, most such patients will be prescribed antibiotic treatment. We are investigating whether treatment of cystitis with an NSAID is as effective as an antibiotic in achieving symptomatic resolution.

**Methods/Design:**

This is a randomized, controlled, double blind trial following the principles of Good Clinical Practice. Women between the ages of 18 to 60 presenting with symptoms of uncomplicated cystitis are screened for eligibility. 500 women from four sites in Norway, Sweden and Denmark are allocated to treatment with 600 mg ibuprofen three times a day or 200 mg mecillinam three times a day for three days. Allocation is conducted using block randomization. The primary outcome is the number of patients who feel cured by day four as recorded in a diary. Adverse events will be handled and reported in accordance with Good Clinical Practice.

**Discussion:**

If treatment of uncomplicated cystitis with ibuprofen is as effective as mecillinam for symptom relief, we can potentially reduce the use of antibiotics on a global scale.

**Trial registration:**

EudraCTnr: 2012-002776-14. ClinicalTrials.gov: NCT01849926.

## Background

Uncomplicated cystitis accounts for 3–4% of all consultations in General Practice and is the most common bacterial infection in women [1],[2]. Half of all women will go through an uncomplicated cystitis during life [3]. Of all these women, 20% will experience recurring infections [4].

Research has shown that if a patient experiences dysuria and pollaksiuria and/or urinary urgency and/or macroscopic hematuria and the symptoms have lasted less than seven days, it is very likely that she has cystitis. If she furthermore has no vaginal symptoms such as discharge or irritation, the likelihood increases to 90% [5]. Hence, it is found that uncomplicated cystitis can be diagnosed and treatment can be prescribed over the telephone [6].

A few trials have been conducted comparing the treatment of uncomplicated cystitis with antibiotics versus placebo. The results showed that the symptomatic improvement and bacteriological cure were significantly delayed in the placebo group, but at the same time the placebo group showed a symptomatic improvement rate of around 50% after three days. In these trials it seemed that most episodes of uncomplicated cystitis were self-limiting and that the patients became symptom free within a week [7]-[9].

In Norway the national guidelines for antibiotic use in primary care recommend a three-day treatment with an antibiotic for uncomplicated cystitis. Mecillinam, nitrofurantoin and trimethoprim are listed as first choices [10]. Previous epidemiological investigations of women with symptoms of cystitis in a general practice population have found *Escherichia coli* (*E.coli*) to be the dominant bacterial agent (70-80%) and secondly *Staphylococcus saprophyticus* (*S.saprophyticus*) (10-15%) [11]-[14]. Retrospective studies have found an increase in bacterial resistance to quinolones, cephalosporins and trimethoprim. Mecillinam and nitrofurantoin still show relatively low levels of resistance to *E.coli* [15].

A pilot study comparing the treatment of uncomplicated cystitis with ciprofloxacin to ibuprofen was recently published. The study included 69 patients and it showed that 58% of the patients who received ibuprofen and 52% of the patients who received ciprofloxacin were asymptomatic after four days. Urine cultures taken on day seven showed more negative cultures among the patients who had received ciprofloxacin (72% vs. 49%), but the difference was not statistically significant [16].

There is an ongoing trial in Germany evaluating whether the use of antibiotics for uncomplicated urinary tract infection (UTI) can be reduced by giving initial treatment with ibuprofen [17].

On a global level antibiotic consumption is on the rise. The rise of consumption and the increase in use of last-resort antibiotic drugs raises serious concerns for public health. Programs that promote rational use through coordinated efforts by the international community should be a priority [18]. The use of cephalosporins, quinolones and long-term use of antibiotics have been identified as risk-factors for infections caused by extended-spectrum- beta-lactamase producing (ESBL) *E. coli* and *Klebsiella species* (*Klebsiella spp*.) [19].

In the US and southern Europe ciprofloxacin has been used as a first choice for treating many infections, including uncomplicated cystitis [20].

The aim of the study is to evaluate ibuprofen versus mecillinam in the treatment of uncomplicated cystitis in healthy, adult, non-pregnant women. Our hypothesis is that symptomatic treatment with NSAIDs is not inferior to antibiotic treatment in this group, and that ibuprofen does not give more complications than mecillinam. Another unanswered question is whether ibuprofen might have an antimicrobial effect.

## Methods

### Design

This is a double blind randomized controlled trial (RCT). Half of the patients will receive treatment with mecillinam and the other half will receive treatment with ibuprofen. The study will follow the principles of Good Clinical Practice (GCP).

### Patients

#### Setting and recruitment

The research project is a co-work between research facilities in Norway, Sweden and Denmark. The study will be recruiting patients from two Accident and Emergency Out Patient Clinics in Norway and general practices in Sweden and Denmark.

#### Inclusion and exclusion criteria

Patients presenting with symptoms of an uncomplicated cystitis will be screened for eligibility using a questionnaire. Such a questionnaire is already in use at the Accident and Emergency Out Patient Clinic in Oslo and we intend to use the same questionnaire at all inclusion sites. The screening will be carried out by a trained study nurse or doctor.

The patients who are eligible will be given information about the study, both orally and in writing. If they decide to participate, they will be given a consent form which will be signed both by the patient and the study nurse or doctor. The consent form is made in accordance with the guidelines of the Regional Committee for Medical and Health Research Ethics in Norway (REK).

Inclusion criteria:

- woman between 18 and 60 years of age
- dysuria and pollakisuria and/or urinary urgency
- ability to give written consent

Exclusion criteria:

- pregnancy or breastfeeding child under one month of age
- diabetes, heart insufficiency, kidney disease or genetic aciduria
- clinical suspicion of pyelonephritis; fever, reduced general condition, upper back pain
- vaginal symptoms such as discharge or irritation
- severe abdominal pain
- symptoms that have lasted for more than seven days
- one or more urinary tract infections within the lasts four weeks
- permanent bladder catheter or use of bladder catheter within the last four weeks
- use of antibiotics within the last two weeks
- participation in a clinical trial within the last four weeks
- previously undergone a pyelonephritis
- previous allergic reaction to penicillin
- previous allergic reaction to ibuprofen, or worsening of asthma when using NSAIDs
- narrow oesophagus
- use of the drug probenecid
- severe gastritis or previous ulcer, Crohn's disease or Ulcerative colitis
- ongoing use of NSAIDs, steroids, anticoagulative treatment or use of immunosuppressant drugs
- thrombocytopenia
- severe psychiatric illness, dementia or severe drug addiction
- unable to communicate in Norwegian, Swedish or Danish language respectively

### Data collection and management

#### Urine samples

Urine cultures are obtained on day one and after two weeks.

#### Symptom score

The patients are given a diary where they daily will register symptom load, possible complications or adverse effects and on which day they feel completely cured. The diary is based on a previously validated version [21]. We will contact the patients after two weeks to make sure they have followed the study procedures. After four weeks we will perform a final telephonic follow up.

### Outcomes

#### Main outcome measures

Proportion of patients who felt cured by day four as registered in the patient diary.

#### Secondary outcome measures

The patients' symptom load with regard to specific symptoms as registered in the diary.

Proportion of patients who had a relapse of symptoms within four weeks after being included in the study.

Proportion of patients who were in need of a secondary medical consultation within the study period.

Proportion of patients who developed an upper urinary tract infection (pyelonephritis).

Proportion of patients who experienced adverse effects and severe adverse effects.

Proportion of patients with a positive urine culture after two weeks.

Evaluation of the use/continued use of the questionnaire in diagnosing uncomplicated cystitis.

#### Does ibuprofen have an antimicrobial effect?

As a sub study it would be of interest to investigate whether ibuprofen has an antimicrobial effect. To do this, it will first be necessary to develop a new test series in the bacteriological laboratory. It is necessary to test breaking points for the antimicrobial effect of ibuprofen in urine samples inoculated with some of the most common urine bacteria such as *E. coli*, *Klebsiella spp*., *S. saprophyticus and Enterococcus spp*..

The details of such a study will be described in a particular protocol at a later stage.

### Sample size

We assume that 85% in the mecillinam group will feel completely cured after four days and consider a 10% absolute reduction to 75% in the ibuprofen group as a maximum relevant difference when holding that ibuprofen is non inferior to mecillinam. With alpha 0.05 and 1- beta of 80% we estimate that we need about 150 patients in each group. To add up for drop outs and withdrawals we aim at recruiting 250 in each group. Stratification is not found to be necessary. We considered whether or not we should expect a cluster effect in the study. We concluded that the cluster effect is negligible as all patients are recruited with the same inclusion and exclusion criteria and randomization is made on the patient level. 85% is based on an ongoing study on uncomplicated cystitis at the Accident and Emergency Out Patient Clinic in Oslo where 85% of patients felt cured after four days. (Marianne Bollestad, personal communication).

### Blinding and randomization

#### Production of the study medicine

The study is double blind. We are buying the original tablets directly from the manufacturers in the pharmaceutical industry, Ibuprofen ratiopharm (ibuprofen) 600 mg from Ratiopharm and Selexid (mecillinam) 200 mg from Leo Pharma. Kragerø Tablettproduksjon AS, a company in Kragerø, Norway, will over-encapsulate the medicine to be used in this study. The original tablets will be put in a gelatine capsule. The capsules will then be filled with small balls of sugar so that the capsules become as similar as possible. The capsule consists of two parts which will be put together and firmly tightened to make it as difficult as possible to open. We have already looked at the final product and this procedure will give the capsules the same look, feel, weight and taste.

The study medicine is packed in two different kits, one with 9 capsules containing one tablet of mecillinam each, the other with 9 capsules containing one tablet of ibuprofen each. Each kit is then labelled with a study number. This labelling is done following a randomization list made by a statistician using block randomization.

At inclusion the patient receives a kit labelled only with the study number. Neither the research nurse or doctor nor the patient will know what active substance is given, thus resulting in double blinding for both nurse/doctor and patient. A list linking the study number to the active substance is kept at Kragerø Tablettproduksjon AS. The list will be retrieved only at the end of the study when all the data has been collected and entered into the statistical program and all analysis are completed.

#### Handling of the study medicine

The study medicine is packed and labelled at Kragerø Tablettproduksjon AS. They will send the study medicine to each of the study sites according to the randomization list. At each study site the study medicine will be handled according to existing local routines and guidelines.

### Intervention

#### Day 1

A nurse or doctor fills out the questionnaire together with the patient. The questionnaire will give us information about symptom load at inclusion. The patient and nurse or doctor sign the consent form.

The patients are randomized into two groups. Both groups receive active treatment and none are given placebo. One group will be given mecillinam 200 mg TID for three days and the other will be given ibuprofen 600 mg 3 TID for three days.

The nurse will take a urine dipstick (leukocytes, nitrite and blood) and register the result in the questionnaire. A urine culture will be sent to the laboratory and the patients will receive equipment to take a new urine sample at home and send it to the laboratory for a second culture after two weeks.

#### Day 1–7

The patients receive a diary where they are going to register the severity of symptoms on a daily basis and on which day they feel completely cured. They will also register possible adverse effects or complications and whether they finished the three day treatment with study medicine or not. If they didn't finish the treatment we will ask how many capsules they have left. When the patients have completed the diary they will send it to the study site.

#### Day 14

The patient will provide a second urine sample for a control urine culture. A study nurse or doctor will call the patients after two weeks for a follow up interview. If they have not yet sent us the diary or delivered a second urine culture, they will be reminded to do so.

#### Day 28

A study nurse or doctor will perform a short telephonic interview with questions about possible relapse of symptoms and need for additional treatment.

The patients will be instructed only to use paracetamol if they have the need for additional pain relief. They are not allowed to take any NSAIDs during the study period as this can interfere with our measurements. If we find they have been taking additional NSAIDs during the study period this will lead to exclusion from the study.

If a patient contacts us having decided to quit the study because she wants to be sure she is given antibiotic treatment, she will be given mecillinam for three days. These patients will be included in the study according to the principle of intention to treat (ITT).

### Safety

#### Criteria for termination of the study

For the individual patient

- developing an upper UTI in need of hospitalization
- serious adverse effect or allergic reaction to the study medicine
- a wish to withdraw from the study

For the study itself

- when the sufficient number of patients have been included
- if more than ten patients within the first 100 patients included are in need of hospitalization, this will be a cause for unblinding of these patients. This is to see whether there are significantly more patients in the ibuprofen group who were in need of hospitalization, and if so we will have to consider terminating the study.

#### Adverse effects

We expect some gastrointestinal adverse effects for both treatments. All adverse effects will be registered in the diary. If the patient should experience an allergic reaction, a severe adverse effect or signs of developing an upper UTI, she is instructed to contact the study centre or the local emergency unit immediately. These conditions demand a change of active treatment and may be a cause to reveal the active substance this patient has been given. A box containing a sealed envelope for each study number will be kept at each study centre. Each envelope, marked only with the study number, contains a note with the name of the active substance given to this patient. If unblinding is necessary, the envelop for this study number will be opened and the patient and the doctor treating the patient will be informed of what active substance the patient has received. The doctor responsible for the study will not be informed and will stay blinded.

When registering the data from the study the adverse effects will be divided into three groups, adverse events (AE), serious adverse events (SAE) and suspected unexpected serious adverse reactions (SUSAR). These will be reported from each study site to the Sponsor using report forms standardized by the Oslo University Hospital according to the principles of GCP.

Detailed information will be sent to the authorities in accordance with Regulations on clinical trials of pharmaceuticals in humans.

#### Insurance

A collective insurance will be taken out through the Norwegian drug liability insurance for all participants in the study. The participants are also insured through the national health service system.

### Registering and handling of data

An investigators study file (ISF) is established at each of the study sites. The ISF will contain all the essential information about the study. A trial master file (TMF) is established in Oslo by the national coordinating investigator. The registering of data will be made consecutively throughout the study period. The data will be registered in a case report form (CRF) for each patient according to the principles of GCP. The handling of the data will follow the principles of GCP. The data will be kept in a locked facility for 15 years after the study is finished. After this it will be destroyed.

### Statistical analysis

Data will be registered and analysed in statistics program SPSS version 22. We will do descriptive analysis and logistic regression analysis.

### Monitoring

A monitor has been appointed in each country. The study will be carried out according to the principles of GCP, the latest revision of the Declaration of Helsinki and national laws and regulations.

### Ethical considerations

Uncomplicated cystitis is predominantly a self-limiting condition and the need for antibiotic treatment of this condition is controversial. However, the condition is often painful and the patients feel the need to take pain medication, often NSAIDs.

In this study half of the patients will receive an antibiotic (mecillinam) and the other half will receive an NSAID (ibuprofen), they will also have the opportunity to take paracetamol in addition to the study medicine, so no one should suffer unnecessary pain.

The risk of developing a more serious infection despite not receiving antibiotic treatment is considered to be small. The pilot study in Germany showed that 33.3% of the patients in the NSAID group and 18.2% in the antibiotic group required secondary antibiotic treatment. The difference was notable, but not statistically significant. None of the patients were in need of hospitalization. No patients experienced severe adverse effects [15].

If the patient qualifies for inclusion in the study she will receive information both orally and in writing. It will be pointed out that participation is completely voluntary and that declining does not affect the kind of help or treatment she will receive. It will be made clear to the patient that she can choose to quit the study at any time. If the patient has any questions regarding the study she can contact a research nurse or a research doctor at the study site. The phone numbers are listed in the consent form received and signed at inclusion.

It will be emphasized that if the patient gets worse during the study treatment or experiences any severe adverse effects, she must contact the study site or come back for a medical consultation. Preferably at the site of inclusion, otherwise at the local emergency unit or other institution with a special agreement with the local study site. Information about such institutions will be made very clear, both orally and in the consent form.

The protocol has been approved by the Norwegian Medicines Agency (NoMA) and by the Regional Committee for Medical and Health Research Ethics. The protocol has also been approved by the corresponding authorities in Sweden and Denmark with minor local adjustments.

## Discussion

As uncomplicated cystitis is the most common bacterial infection in adult women, this represents a large portion of the consultations at a general practice. If we could reduce the need for antibiotic treatment for this condition, and maybe find that it can be treated with a simple over-the-counter drug such as ibuprofen, this will reduce the number of consultations and the total use of antibiotics.

A double blind RCT ensures reproducibility of the results. In accordance with national guidelines for diagnosis and management of acute uncomplicated UTIs, inclusion was not determined by bacteriologically verified UTI, but by typical symptoms [22].

Bacteriological samples are secured at the time of inclusion, in the case of treatment failure and after finishing treatment. This allows for analysis of relation between treatment failure and bacteriological findings, as well as allowing for a sub study of antimicrobial effect of ibuprofen.

If we demonstrate that the proportion of patients with persistent bacteriuria is equal in the two groups, this will show that the immune system of the women in the ibuprofen group may have handled the bacteria without antibiotics, or that ibuprofen may have an antimicrobial effect.

If we find that and NSAID is equally effective as an antibiotic, this may contribute to a reduction in the use of antibiotics and reduce antibiotic resistance globally. This is beneficial to the environment and will reduce the costs in health services internationally.
